# Stricter Control

**Published:** 1890-06-21

**Authors:** 


### Passing Topics.

### "STRICTER CONTROL."

Those who give evidence before the Select Committee of the House of Lords, must expect to find that their utterances are not always acceptable to those with whom they would desire to be in accord. Mr. Robert Bousfield must be experiencing the truth of this observation. Whatever else his acquaintance with hospital work may have taught him he cannot have failed to learn that it is essential to the well-being of a hospital that the relations of the lay and medical authorities should be harmonious, and it is impossible to suppose that when he told the Committee he thought "it would be better if the control over the medical staff was stricter," he meant anything calculated to give offence.

But Dr. Lionel Beale is not satisfied of this, and with the instincts of a true hospital physician, grows restive at the merest hint of such things as duty and control, and writing with some excess of warmth to the *Lancet* claims absolute freedom for himself and his colleagues.

Probably, no one is more surprised than Mr. Bousfield at the reception his piece of evidence has met with, yet he might have known that of all dangerous ground which a lay manager of a hospital can tread none is so dangerous as that he has ventured upon in his effort to fix this vague generality. Stricter control for hospital staffs indeed! No wonder that Dr. Lionel Beale uses a note of exclamation after the portentous sentence. The base suggestion arouses all the indignation of a profession which brooks no control but of its own making, and is not always prepared to accept that.

Do not all of us know that while the rest of the world advises or recommends, the doctor orders? Where is the professor of medicine worthy of his stethoscope who would obey the behests of laymen, however exalted, and the correspondent of the *Lancet* may well inquire, with a fine intermingling of scorn and reproach, whether Mr. Bousfield holds "the intolerable theory that the members of the medical staff are the servants of the committee?" We cannot say that anything he is reported to have uttered shows that he does. There is a very considerable difference between being a servant of the committee and being held to the performance of the definite duties a hospital physician undertakes in return for certain well-recognised and valuable advantages. No sensible man would for a moment propose that a lay authority should be allowed to exercise any sort of control over the members of a hospital staff, either in regard to the treatment of their several patients or whatever is cognate to their profession. But the representatives of those who provide the funds and have all the anxieties incidental to the general management and maintenance of an institution may reasonably ask to be allowed to take steps to ensure that its chief obligations should be rightly discharged.

The "stricter control" which was doubtless in Mr. Bousfield's mind, and which, we venture to think, will certainly be obtained some day, need not be a "faddy, fidgety interference"—it will be fatal to the best interests of the patient if it is; but simply the power to ensure that duties undertaken shall be faithfully and punctually performed, and not alone for the sake of the profession and of science but with a view to the utmost good of the patients.

No one intimately acquainted with hospital life can doubt that most of the physicians and surgeons perform their duties with exemplary exactitude and humanity, but it is undeniable that some neither set the same value upon the opportunities afforded them, nor estimate fully the philanthropic element in the work the hospital undertakes. It is a strange contention that there shall be no authority to quicken a sense of duty when necessary, or to relieve the institution of the permanently negligent officer. At present this is the position some hospitals are placed in. Agricola.

